# Charter of the College of Physicians and Surgeons

**Published:** 1888-06

**Authors:** 


### THE CHARTER OF THE COLLEGE OF SURGEONS.

The official announcement made by Sir W. Hart Dyke in the House of Commons, and the report which we publish in another column of the proceedings of the Council of the College of Surgeons, will afford to the profession what is probably to the bulk of our readers the sensation of a painful surprise. The Privy Council, under the well-devised official formula of declining to deal with contentious matter, have practically refused off-hand the applications of the fellows and members for amendments of the charter. There are probably few precedents for dealing in this summary way with a petition of so many thousands of the members of a college for the better recognition of their rights in their own corporation. The Privy Council, or rather the one or two distinguished gentlemen who act for it in these matters, received in this case a petition of almost unexampled numbers and weight from the educated members of a professional body, and a further formal request from a delegated deputation that if their claims were not admitted they might be heard by counsel in support of them. Nevertheless, it now appears that, without reply from the existing authorities of the college, and even without the usual courtesy of any further communication with the association of members, the advisers of the Queen have summarily snuffed out their claim. Such a course of action calls for immediate and energetic protest. The claims of the members may be right or wrong, but no steps have been taken to convince them that they are unfounded either in policy, justice, or law. No answer of any sort has been vouchsafed to their arguments or their claims, although these were based, among other things, upon the strongest allegations as to the true constitution of the body whose charters were in question, and showed strong reasons for the belief that many matters in the existing organization amounted practically to an abuse.

The Privy Council cannot have convinced itself either of the impolicy or of the want of legal basis for these contentions, since it neither received any answer in respect to them from the promoters of the charter nor afforded any opportunity for a judicial or quasi-judicial consideration of the issues involved. Trusting to the usual fairness and courtesy of great official bodies in such matters, no discussion has meantime been raised in Parliament, and at this period of the session it is difficult, if not impossible, to secure the attention of Parliament to the subject in any effective manner until next February. It is perhaps undesirable that the members should be driven to enforce the rights they claim by any litigation with their own college, and especially so as these claims are much more largely based upon questions of public policy and general justice than of technical legal right, although the latter would appear not to be wanting.

Even yet, however, it is not too late for Her Majesty's advisers to reconsider their present attitude towards the question. It would appear to be still open to the members to address an immediate petition to the court praying that the matter of such petition and of the previous petition signed by 6,000 members of the college already before the Privy Council, may be remitted to the Privy Council in its judicial capacity for further debate in order that they may advise the crown with judicial responsibility, after hearing such evidence as the parties interested may desire to produce and the arguments of counsel thereon. We believe that there is nothing contrary to constitutional practice in such a course, although precedents of this precise character are, of course, rare, as are the circumstances which lead to it. It is highly unsatisfactory that so important a question should be burked by an official body, which practically sets aside a claim the equity of which it neither discusses nor decides. It must not be forgotten that even the limited supplemental charter which it is now proposed to grant will perpetuate and increase that autocratic form of administration against which the bulk of the members have petitioned and protested, by empowering the existing authorities to hold and administer even a larger amount of corporate property than that which they already possess. The members can hardly allow the matter to rest here, if they are well advised and properly led.—*British Medical Journal.*

